# CT Scan and Clinical Outcomes of Novel Lateral-Oblique Percutaneous Sacroiliac Joint (SIJ) Fixation: Technique and Literature Review

**DOI:** 10.7759/cureus.16408

**Published:** 2021-07-15

**Authors:** Kingsley R Chin, Fabio J Pencle, Jason A Seale, Deepak K Pandey

**Affiliations:** 1 Orthopedic Surgery, Less Exposure Surgery (LES) Clinic, Hollywood, USA; 2 Orthopedic Surgery, Florida International University, Herbert Wertheim College of Medicine, Miami, USA; 3 Sports Science, University of Technology, Kingston, JAM; 4 Orthopedics, Less Exposure Surgery (LES) Clinic, Hollywood, USA; 5 Orthopedics, Less Exposure Surgery (LES) Society, Malden, USA

**Keywords:** percutaneous lateral-oblique, sacroiliac joint fixation, sacrix line, mini-open, compression screw, sacroiliac displacement, dislocation

## Abstract

Purpose

The sacroiliac joint (SIJ) is estimated to be a source of pain in 15%-30% of patients presenting for the evaluation of low back pain. The SIJ may develop symptoms in an estimated 43% of patients who have had previous lumbar fusion surgeries. With increased awareness of SIJ as a pain source and for those patients who have intractable pain and who fail nonoperative treatment, surgery to stabilize the SIJ is becoming more common. Thus multiple different technologies and techniques need to be evaluated. The purpose of this study is to report on the clinical and radiographic follow-up of percutaneous lateral-oblique sacroiliac joint fusion with a threaded compression screw performed in an outpatient ambulatory surgery center (ASC).

Methods

Three consecutive patients were chosen for this technique, and after completion, were followed for at least 24 months as part of a pilot study to see how they responded to the treatment. The medical charts of these patients were reviewed along with follow-up radiographs and computed tomography (CT) scans to assess for radiographic fusion designated as bridging bone across the SIJ with no signs of implant loosening such as haloes around the screws, change in position, or screw breakage. The SacroFuse (Sacrix LLC, Boston, MA) SIJ screws were 12 mm x 60 mm at S1 and 12 mm x 50 mm at S2 with threads for compressive fixation and cannulated for percutaneous placement over a guidewire. We evaluated patients' demographics, the pain visual analog scale (VAS) score, and the Oswestry Disability Index (ODI) preoperatively and postoperatively.

Results

Our first patient was a 51-year-old male body mass index (BMI) 33.3 kg/m^2^ with a previous lumbar fusion. He underwent a two-staged SIJ fusion. The first surgery was done as an open direct lateral surgery, and the second stage was performed three months later using a direct percutaneous lateral-oblique technique for three months. The second and third patients, respectively, were 22-year-old female status prior L5-S1 anterior lumbar interbody fusion (ALIF) plus right posterior unilateral pedicle screws. She had a BMI of 38.3 kg/m^2^. The third patient was a 41-year-old male with a BMI of 29.5 kg/m^2^ who underwent lateral-oblique bilateral percutaneous SIJ fixation. The latest CT imaging of each patient demonstrated increased bone density adjacent and within implants with intra-articular osseous bridging. There were no implant failures or complications.

Conclusion

This pilot study demonstrated the feasibility and effectiveness of a new percutaneous lateral-oblique SIJ fusion technique with a threaded compression screw done safely in an ASC. Patients demonstrated early pain relief and long-term fusion of their SIJ. We introduced the Sacrix line as a key fluoroscopic landmark for the success of this percutaneous technique.

## Introduction

The sacroiliac joints (SIJs) are the largest axial joints in the body, connecting the sacrum to the ilium of the pelvis. Motion in the SIJ is limited mainly to rotation around the S2 axis, more specifically called nutation and counter-nutation, because of the sinusoidal pattern (as opposed to a spherical pattern) [1]. The SIJ is increasingly recognized as a common source of chronic low back pain. The SIJ may play a role in 15%-30% of patients presenting for evaluation of low back pain [2-6]. The SIJ is commonly suspected (up to 43%) as a source of pain in patients who have undergone prior lumbar fusion [7-8]. This can be attributed to highly invasive, adjacent open operations with long hospital stays, recovery times, high nonunion rates, and poor long-term satisfaction rates [7- 8]. Studies have confirmed the safety and radiographic fusion of SIJ fusion [9-10]. In a study by Spain et al., SIJ fixation with screws had a higher revision rate than triangular titanium implants [11]. Limited studies are available demonstrating fusion in the lateral-oblique approach compared to the lateral MIS approach. The authors aim to present the clinical outcomes and radiographic evidence of fusion using this technique.

## Materials and methods

**Figure 1:**
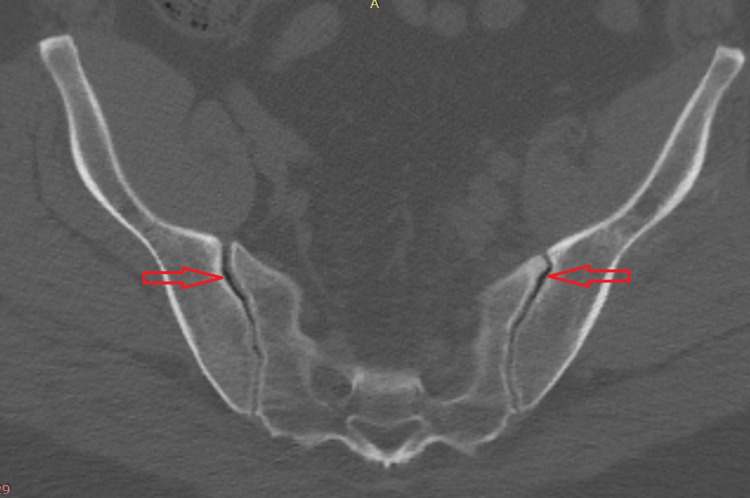
Preoperative CT scan showing bilateral SIJ vacuum sign CT: computed tomography; SIJ: sacroiliac joint

**Figure 2:**
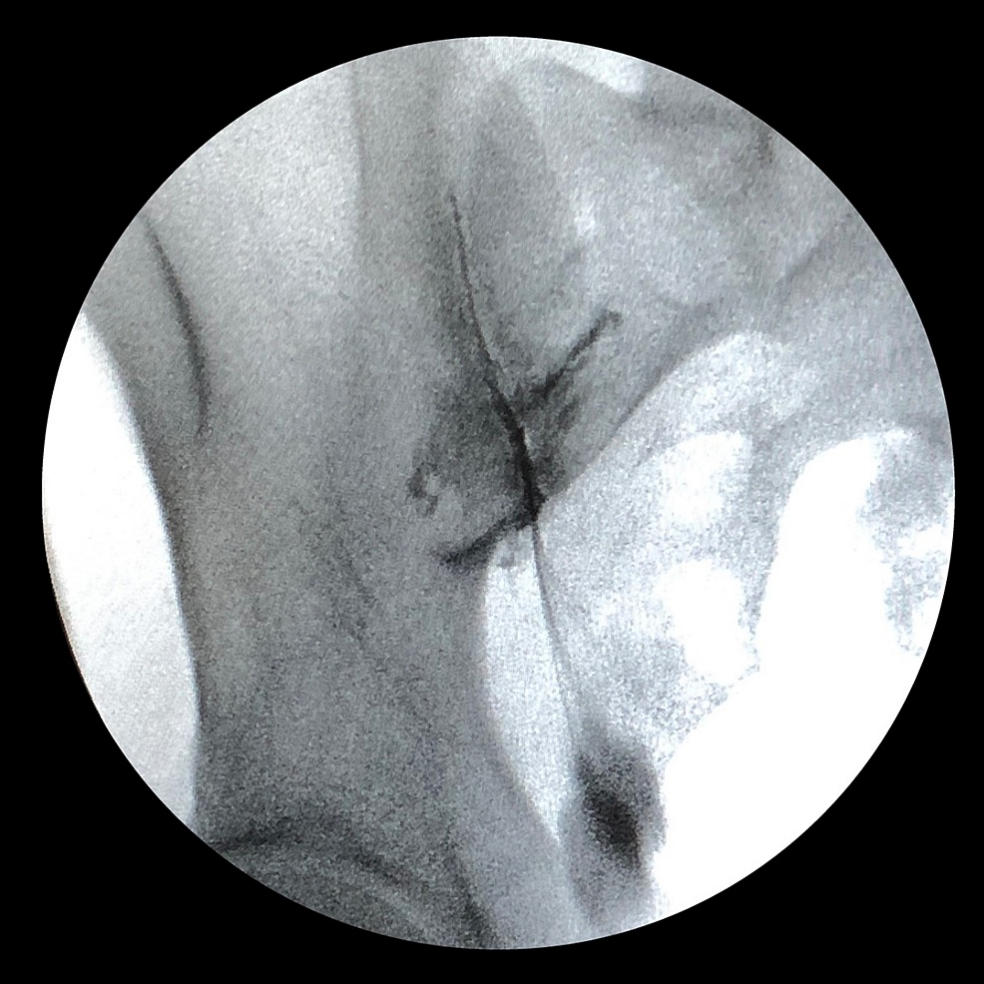
Diagnostic fluoroscopy demonstrating SIJ contrast test SIJ: sacroiliac joint

This article demonstrates a technique for bilateral percutaneous lateral-oblique sacroiliac fixation. Inclusion criteria for surgery included physical examination, imaging, and failed conservative management (medication or steroid joint injection). Clinical testing included three out of five positive provocative tests: distraction, thigh thrust, compression, Gaenslen's, and flexion, abduction, external rotation (FABER). Imaging included preoperative X-rays and MRI/CT (Figure 1) and SIJ dye testing (Figure 2).

Prior to surgery, all patients signed an informed consent form that explained the procedure, risks, and benefits and were informed of data collection for research purposes. Patients had the opportunity to withdraw their consent at any time. All procedures were performed as part of the standard of care and did not involve any investigational procedures or products. Patient data were collected at baseline, six weeks post-surgery, and then at three-month intervals up to two-year post-procedure. Data regarding these groups were collected from medical records and operative notes. Institutional Review Board (IRB) approval was granted through protocol # 20181251.

Video 1 shows the percutaneous lateral-oblique surgical technique.

**Video 1:** Video showing fluoroscopy steps and markings

Under general anesthesia, the patient was placed prone on a Wilson frame in a neutral position. The patient’s lower lumbosacral area was prepped and draped in the usual sterile fashion. Fluoroscopic anteroposterior (A-P) views, 30-degree oblique views, and 15-degree pelvic outlet tilt views were obtained to identify the anatomy and important landmarks. To begin, the fluoroscopy was placed in a 30-degree oblique view. A 15-degree pelvic outlet tilt view position, which gave an excellent view of the Sacrix line (lateral border of the ilium), first sacral vertebrae (S1) endplate, the teardrop (where the Sacrix line merges with the anterior superior iliac spine), and the superior and inferior sacral alar was obtained. This also gave us excellent visualization of the sacroiliac joint. Using a guidewire, a vertical skin marking corresponding to the Sacrix line was drawn. Parallel horizontal skin markings were made corresponding to the superior and inferior alar. 0.5% marcaine with epinephrine was injected approximately 2 cm lateral to the Sacrix line and within the alar skin markings. Under direct hand control, a Jamshidi needle was placed through a small stab incision. The tip of the Jamshidi needle was used to feel the top of the iliac crest. The Jamshidi needle was then advanced at a 10-20 degree mediolateral angle until it crossed the sacroiliac joint and into the alar by approximately 2 cm under fluoroscopy guidance. The Jamshidi has a beveled tip to allow directional control away from the beveled side. The screw trajectory was assessed using a lateral fluoroscopy view. The S1 screw trajectory was aimed towards the tip of the S1 sacral promontory and below the L5-S1 disc space. The inner trocar was removed, and a blunt nitinol guidewire was placed through the needle until it passed the tip of the Jamshidi needle and was secured into the bone. We removed the Jamshidi needle and made a 1.5 cm skin incision. We inserted the dilator and working cannula over the guidewire. Once the working cannula was docked, we removed the dilator and confirmed the position on fluoroscopy. We tapped over the guidewire with a 9 mm sized tap. At S1, a 12 mm x 60 mm cannulated screw with threads for compressive fixation (SacroFuse, Sacrix LLC, Boston, MA) was prepacked with NanoFuse Biologics Putty (NanoFuse Biologics LLC, Malden, MA). We inserted each Sacrofuse screw over the guidewire and threaded it across the SIJ into the sacral ala until we had great fixation. Identification of the pelvic rim using a 45-degree pelvic inlet view confirmed screw depth. We repeated the procedure for the placement of a second screw at S2. The Jamshidi needle was placed parallel to the first screw using the oblique tilt and pelvic inlet fluoroscopic views. This allowed us to place a 12 mm x 50 mm cannulated screw at S2 using the same incision. If the patient has bilateral SIJ symptoms, the procedure was repeated on the contralateral SIJ. The wound was closed in layers.

Follow-up was at six, 12, and 24 months, with outcomes scores assessed and CT scan performed at the 24-month follow-up. An evaluation was performed by three separate radiologists who assessed fusion relative to the implant and anatomy of the joint.

Statistical analysis

We performed statistical analysis with SPSS v22 (IBM Corp., Armonk, New York). Tests were considered significant if p<0.05.

## Results

The first patient was a 51-year-old male with a body mass index (BMI) of 33.3 kg/m^2^. The procedure was performed in two stages. The first stage was direct open lateral; the second stage was three months after open lateral-oblique.

The second patient was a 22-year-old female post L5-S1 anterior lumbar interbody fusion (ALIF), L5-S1 fusion, with a BMI of 38.3 kg/m^2^. The patient’s procedure was a percutaneous lateral-oblique bilateral SIJ fixation. The third patient was a 41-year-old male with a BMI of 29.5 kg/m^2^ with percutaneous lateral-oblique bilateral SIJ fixation.

Preop mean visual analog scale (VAS) scores 8.33+/-1.53 decreased to 2.33+/-0.88 and showed a statistically significant difference (p=0.0086). Preop mean Oswestry Disability Index (ODI) 62+/-1.15 decreased to 19+/-2.31 (p=0.001). Preop mean 12-item short-form survey (SF 12) physical component summary (PCS) score of 25.53+/-1.8 increased to 52.32+/-0.8 (p<0.0001), mental component summary (MCS) score of 26.8+/-3.7 increased to 56.3+/-1.7 (p=0.0021). Demographics and outcome results are shown in Table 1.

**Table 1:** Demographics BMI: body mass index; Pre: preoperative; Post: postoperative; VAS: visual analog scale; ODI: Oswestry Disability Index; SF: short-form survey 12 item; PCS: physical component score; MCS: mental component score

	Patient 1	Patient 2	Patient 3	Mean
Age (years)	51	22	41	31.50
BMI (kg/m^2^)	33.3	38.3	29.5	33.70
Pre VAS	8	7	10	8.33
Post VAS	4	2	1	2.33
Pre ODI	64	62	60	62.00
Post ODI	23	19	15	19.00
Pre SF PCS	25.62	27.29	23.69	25.53
Pre SF MCS	28.32	32.4	19.68	26.80
Post SF PCS	52.22	51.55	53.18	52.32
Post SF MCS	53.63	55.58	59.7	56.30

**Figure 3:**
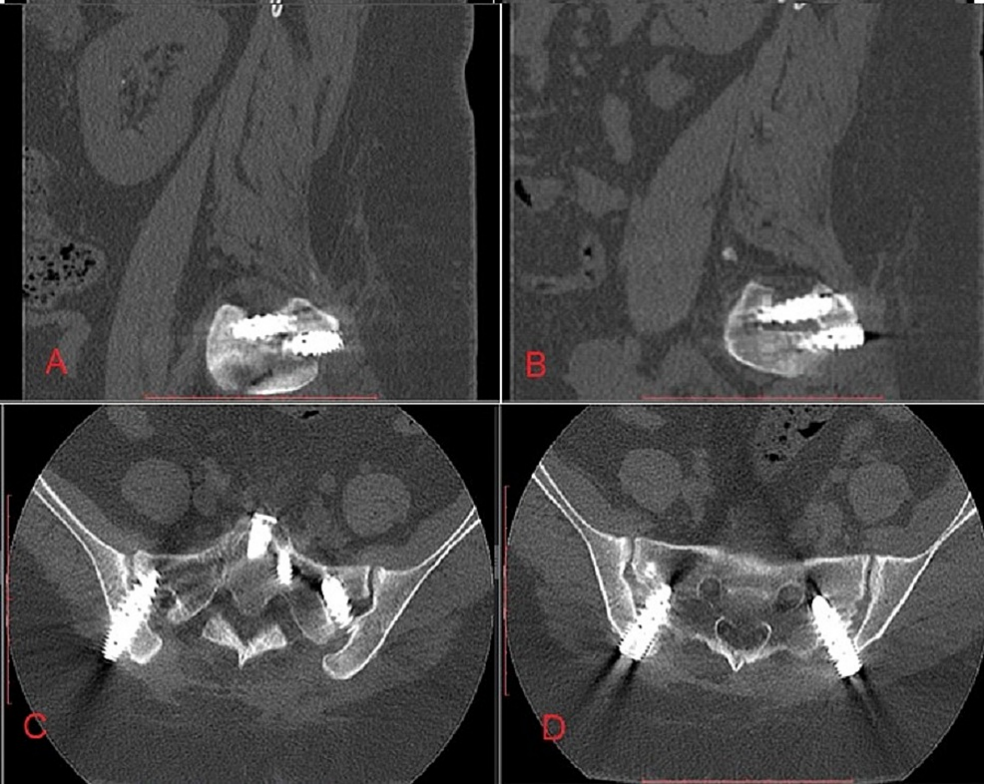
Postoperative CT scan showing fusion A: Sagittal slice left; B: Sagittal slice right; C: Axial slice S1 vertebrae; D: Axial slice S2 vertebrae

CT scans of the pelvis with axial, sagittal, and coronal reconstructions documented increased bone density circumferentially adjacent to the implant and within the implant. Figure 3 shows intra-articular osseous formation with bridging bone at the anterior and posterior aspects of the joint. Table 2 summarizes surgical procedure, operative time, blood loss, and fusion success with a total follow-up. 

**Table 2:** Summary of operative times, surgical procedure and follow up period Mins: Minutes mls: milliliters

Patient	Operative time (mins)	Blood Loss (mls)	SIJ Procedure	Fusion Success	Follow Up Period
1	65	70	Left Open Lateral Fixation	Fused	32 months
1	50	40	Right Open Direct Lateral-Oblique Fixation	Fused	32 months
2	34	50	Percutaneous Lateral-Oblique Bilateral Fixation	Fused	24 months
3	37	35	Percutaneous Lateral-Oblique Bilateral Fixation	Fused	24 months

## Discussion

The indication for sacroiliac joint fusion includes back pain when sitting or lying, dull ache below the waist, buttock pain that may radiate to the thigh/groin, and pain while climbing stairs or hills. Popular techniques to perform sacroiliac joint fusion are lateral techniques to fuse the joint via a triangular peg or screw [12-13]. The use of posterior techniques to fuse the joint via screw or to distract the joint by bone plug has also been demonstrated [14-15]. The authors aimed to demonstrate the percutaneous lateral-oblique technique for sacroiliac fusion using SacroFuse by Sacrix. The percutaneous lateral-oblique technique offers an advantage over the lateral technique. It provides easier access to the surgical site, as the device goes through less tissue and muscle, and is less invasive due to less muscle disruption during surgery. All the patients experienced improved quality of life, pain, and function after surgery, which did not occur with nonsurgical management. SIJ fusion has been demonstrated by Whang et al. [16] and Polly et al. [7] in a randomized control study as being more effective for the treatment of SIJ pain for six-months and one-year follow-up. Martin et al., looking at the current evidence of minimally invasive SIJ fusion, assessed the studies for the lateral transiliac approach and the dorsal approach [17]. Eleven Level 4 triangular implant studies had a total of 361 patients. The largest studies by Sachs et al. in 2014 [18] and 2016 [19] with one implant malposition and five revision surgeries, respectively. Wise and Dall demonstrated up to 89% fusion in a six-month prospective study [20].

A retrospective study of 20 patients by Beck et al. [21] assessed the procedure satisfaction rating of sacroiliac fusion. All patients had a positive SI joint injection with a mean age of 57.7 years. They used two techniques for mini-open arthrodesis. The first technique was a posterior medial oblique approach; however, the technique was changed to a direct trans-cleft approach due to the anatomy. The trans-cleft approach is similar to our percutaneous technique. The average patient satisfaction rating was 7.25, with a 95% fusion rate. The patient who did not achieve fusion was due to the cage being removed, as it was incorrectly placed [21].

Our study demonstrated improved ODI and SF-12 outcomes with fusion in all three patients. We demonstrated the change in approach from lateral to percutaneous lateral-oblique with fusion being achieved at the end of the two-year follow-up.

The authors acknowledge this technical guide’s limitation of a small sample size. We, however, note the technique is the first to demonstrate the use of the iliac crest and the Sacrix line as landmarks for the surgery.

## Conclusions

Our paper demonstrates the surgical technique of transitioning from direct lateral to percutaneous lateral-oblique sacroiliac fixation. A key step included identifying the Sacrix line using a 30-degree oblique view and a 15-degree pelvic outlet tilt view on fluoroscopy to square off the S1 endplate and visualize the SIJ. To evaluate the final screw placements, we recommend a 45-degree pelvic inlet view to evaluate the screw depth in relation to the pelvic rim.
